# Identification by the Teeth

**Published:** 1890-09

**Authors:** 


IDENTIFICATION BY THE TEETH.—On the 21st of July last a dead body was found floating in the Potomac river above Georgetown, D. C. About one week previous, Charles C. Andrews, formerly of Baltimore City, but at the time living in Washington, D. C., disappeared from his home and had not been seen since that time. A few days ago the body found in the Potomac was identified by Dr. T. W. Coyle, a practicing dentist of Baltimore, as that of Mr. Andrews by the dental work done by Dr. Coyle, in June 1876. The following is the record of the dental work and a history of the case:

Mrs. Andrews, wife of the missing man, went to Dr. Coyle's office, 825 North Eutaw Street, and asked that he go over to Washington to examine the body of the man found on July 21, and see if he could recognize it. It was impossible at that time for Dr. Coyle to go, and yesterday Frank Andrews brother of the missing man, Detective McDevitt, of Washington, the representative of an insurance association, and two others, took to Dr. Coyl's office the skull of the drowned man. As soon as he looked at the jaws he recognized the skull as that of Charles C. Andrews by the fillings he had put in the teeth fourteen years ago, when Andrews was a young man of twenty-four. The greater part of the work was done on June 26, 1876. At that time, and subsequently, Dr. Coyle inserted in the upper jaw of Andrews a filling in a crown cavity in the right twelve-year-old molar, an approximal cavity in the right lateral incisor, a lateral approximal cavity in the left eye tooth, a crown cavity in the left posterior bicuspid and a crown cavity in the left wisdom tooth. He extracted three of the front teeth, one right anterior bicuspid, and there were teeth on the left side out, a molar and two bicuspids. He made a set of seven false teeth on a rubber plate all for the upper jaw. In the lower jaw he filled a number of teeth which he remembered. All of these fillings were of gold except the last which was of amalgam. The set of false teeth were gone, as was the left eye tooth in the upper jaw. The loss of this tooth was due to the handling of the body, the examinations that have been made &c. One of the men present, who saw the body when found says the tooth was in its place then. Dr. Coyle rummaged among a lot of his old books and at last found that containing some of the accounts of his patients for the year in which he did work for Andrews. Over Andrews's account were exact diagrams of his mouth and of the work Dr. Coyle had done for him. Every one present was perfectly satisfied after Dr. Coyle had explained the diagrams that the head was that of Andrews, and Dr. Coyle says he is willing to make oath to it before any court in the land. He is satisfied that the body could have been identified in no other way. All the fillings were in place, as sound and solid almost as the day they were put in. When the body was found in the Potomac, near Georgetown, D. C., it was entirely nude, and no trace of clothing has yet been found. It was buried in the almshouse cemetery, it was exhumed once after that for identification, then reinterred, and then within the last day or so exhumed again in order that the skull might be brought to Dr. Coyle. Mrs. Andrews has claimed the body, which will now be prepared for burial again and brought to Baltimore and interred in

Bonne Brae Cemetry on Saturday. Mrs. Andrews is now perfectly satisfied that the body is that of her husband. She has believed it to be for some time past, though she could not identify it because of decomposition. The police, who are investigating the mystery of his death, are as yet without a clue.

An article in the Baltimore *Daily Sun* relating to questions of "dental jurisprudence," contains the following:

The remarkable identification of the body of Charles C. Andrews by Dr. T. W. Coyle, the Baltimore dentist, after the finding of the body in such a condition of nudity and decomposition that identity was not possible by ordinary means revives the question of dental jurisprudence. It was in 1883 that Richard Grady, M.D., D.D.S., made a plea before the Maryland and District of Columbia Dental Association that "Dental jurisprudence should have not only a name, but a local habitation." In Baltimore, the oldest and most widely known American centre of dental learning, has occurred the most striking evidences of the value of dental jurisprudence, not alone in the Andrews identification, but Dr. Grady showed in the Goss-Udderzook murder case. The Doctor noted in his paper twenty cases of personal identity that were established by the teeth, some of which have a local interest here.

His paper says: "The Goss-Udderzook tragedy was a double story of fraud in the earlier stage and murder in the later. February 3, 1872, a newspaper stated that W. S. Goss, living at 314 North Eutaw St., had been burned to death in a cottage outside of Baltimore the previous night. The remains of a human body were taken from the building. The lower limbs were destroyed, and the features were so burned or charred as to be beyond recognition. From the shape of the chest, neck and head the corpse was identified to be that of W. S. Goss, so the coroner's jury rendered a verdict that W. S. Goss came to his death by the explosion of an oil lamp. The body was buried at Baltimore Cemetry. The widow had no question but that it was that of her husband, as she knew the contour of the neck, head and breast. Ten or more witnesses testified to their belief in the some identity.

"In May, 1871, W. S. Goss had seemed to be seized with a sudden mania for insuring his life. He had insurance amounting to $25.000 payable to his wife. The stories of Wm. E. Udderzook, a brother-in-law of Goss, cenveyed the the impression that he knew too much and led the insurance company into an inquiry. There was nothing in the way of a direct demonstration to show fraud. Mrs. Goss was asked to make an elaborate description of her husband's teeth which she said were solid, sound and regular. She said that during the fourteen years she had lived with him he had had no pain from his teeth and had not required the services of a dentist.

"The remains were exhumed and examined in the Baltimore College of Dental Surgery, and Prof F. J. S. Gorgas wrote a report and modeled plaster casts of the jaws, showing that the buried man must have had teeth drawn frequently by a dentist, and that his teeth could never have been truthfully called regular and sound. In a trial in which the companies attempted to prove that the body found was not that of Goss, Dr. Robert Arthur testified that the plaster model of the subjects mouth, made by Dr. Gorgas, showed much disease, and much pain had been suffered. The jury returned a verdict on June 26, 1878, for Mrs. Goss for the amount of insurance, with interest. On July 1 or 2 the body of Goss, who, had been murdered, was found in Chester county, Pa., after Udderzook had been with him. The description of the body included the teeth, and Udderzook was convicted of murder in the first degree."

Another case is that of Miss May Hatch, given as follows: "Miss Hatch, of Baltimore, went to Norfolk, Va., June 16, 1888. There she took the steamer for Boston, and when on the ocean it is claimed committed suicide by drowning. To perfectly establish the identity of the remains Dr. Norris, of Baltimore, examined the teeth, and comparison with his diagrams left no doubt regarding the identity of the corpse." In addition to these cases are cited the identification of C. Arthur Preller, the victim of the St. Louis trunk mystery, and that of the Prince Imperial of France, who was killed in Africa.

